# Long-Term Survival Analysis of Transarterial Chemoembolization Plus Radiotherapy vs. Radiotherapy for Hepatocellular Carcinoma With Macroscopic Vascular Invasion

**DOI:** 10.3389/fonc.2020.01205

**Published:** 2020-07-31

**Authors:** Ting-Shi Su, Li-Qing Li, Wan-Wan Meng, Yu-Dan Wang, Yi-Tian Chen, Jian-Xu Li, You-Qin Du, Song Qu, Chang Zhao, De-Jia Huang, Shi-Xiong Liang, Le-Qun Li

**Affiliations:** ^1^Department of Radiation Oncology, Guangxi Medical University Cancer Hospital, Nanning, China; ^2^Department of Interventional Radiology, Guangxi Medical University Cancer Hospital, Nanning, China; ^3^Department of Hepatobiliary Surgery, Guangxi Medical University Cancer Hospital, Nanning, China

**Keywords:** hepatocellular carcinoma, major vessel invasion, TACE, radiotherapy, overall survival, radiation-induced liver disease

## Abstract

**Background:** Macroscopic vascular invasion (MVI) is a terminal manifestation of hepatocellular carcinoma (HCC) and carries an extremely poor prognosis. In Chinese and Korean HCC guidelines, transarterial chemoembolization (TACE), or/and radiotherapy (RT) is adopted for treatment of MVI. In the current study, we aimed to compare the long-term outcome of TACE + RT to that of RT alone in patients with local advanced HCC with MVI.

**Methods:** In this retrospective study, 148 treatment-naive patients of HCC with MVI were enrolled. Of the patients enrolled, 49 received TACE + RT treatment, whereas 99 patients received RT alone as a monotherapy. Overall survival (OS), progression-free survival (PFS), and intrahepatic control were evaluated using univariable and propensity score–matched analyses.

**Results:** During follow-up, 126 patients (85.1%) died. The median follow-up time was 55.0 months in the RT group and 57.0 months in the TACE + RT group. The TACE + RT group showed better OS and PFS than the RT group, but intrahepatic control was comparable in these two groups. Of 41 cases well-pairs after propensity score matching, the associations between TACE + RT and better OS and PFS remained (15.0 vs. 8.0 months, and 8.0 vs. 4.0 months, all *P* < 0.05). The 1-, 2-, 3-, and 5-years OS rates in the TACE + RT group were 56.1, 28.6, 20.8, and 15.7 vs. 31.5%, 13.1%, 9.8%, and 6.7% in the RT group, respectively (*P* = 0.017). The 6-, 12-, and 24-months rates in the TACE + RT group were 51.2, 39.0, and 23.1% vs. 36.6%, 13.9%, and 11.1% in the RT group, respectively (*P* = 0.04). Two patients (4.1%) experienced radiation-induced liver disease (RILD), and one (2.0%) experienced RT-related gastrointestinal (GI) bleed in the TACE + RT groups. Nine patients (9.1%) experienced RILD, and two (2.0%) experienced RT-related GI bleed in the RT groups.

**Conclusion:** Transarterial chemoembolization + RT had well-complementarity with no more complications than RT alone, providing a better PFS and OS compared with RT-alone treatment for HCC with MVI.

## Introduction

The World Health Organization estimates that more than one million patients will die of liver cancer in 2030 on the basis of annual projections (1). Hepatocellular carcinoma (HCC) accounts for the majority of primary liver cancers and is characterized by a strong propensity to invade the surrounding hepatic vasculature (2, 3). Macroscopic vascular invasion (MVI) involving portal vein tumor thrombosis (PVTT) and/or hepatic vein tumor thrombosis (HVTT) and/or inferior vena cava tumor thrombosis (IVCTT) is recognized as a common accompanying manifestation in patients with advanced HCC, with 44–84% in HCC patients from autopsy data (4, 5) Macroscopic vascular invasion is a bottleneck in the treatment of HCC. Controversy exists from the West and the East on the treatment of these patients. Western European Association for the Study of the Liver (EASL)–European Organization for Research and Treatment of Cancer practical guidelines, which are based on the Barcelona Clinic Liver Cancer (BCLC) staging system, consider HCC with MVI to be at the advanced BCLC stage C, and sorafenib is the only evidence-based treatment option for this patient group (6). In Chinese and Korean guidelines, transarterial chemoembolization (TACE), surgery, systemic treatment, and radiotherapy (RT) were more frequently adopted for treatment of selected HCC patients (7–9). Recently, a randomized clinical trial showed that first-line treatment with TACE + RT was well-tolerated and provided improved survival outcomes compared with sorafenib treatment for patients with MVI (10). The results following RT alone or TACE and RT were better compared to untreated controls or those treated with TACE alone (11–14). However, few comparative studies have analyzed the use of TACE + RT vs. RT alone in patients with MVI, to our knowledge. In this retrospective study, we sought to compare the long-term survival outcome of TACE + RT to RT alone for these selected patients with HCC.

## Materials and Methods

### Patients

From 2000 to 2016, 148 HCC patients with MVI treated with RT alone or as an adjunct to TACE in Guangxi Medical University Cancer Hospital were enrolled. Eligibility criteria were as follows: (a) HCC was diagnosed by histopathology or criteria according to the EASL Clinical Practice Guidelines for the management of HCC (6); (b) the presence of medically inoperable MVI was diagnosed based on characteristic imaging findings (hypodense filling defect for portal, hepatic, and/or inferior vena cava vein thrombus) obtained by four-phase computed tomography (CT) scan and/or dynamic contrast-enhanced magnetic resonance imaging (MRI); (c) Child–Pugh class A or B disease; (d) Eastern Cooperative Oncology Group (ECOG) score of 0–1. Exclusion criteria were as follows: (a) Child–Pugh class C disease with poor liver function; (b) ECOG ≥ 2; (c) extrahepatic metastasis; (d) underwent concurrent treatments, including systemic chemotherapy, or molecular targeted therapy. The study was approved by the Institutional Ethics Committee of Guangxi Medical University Cancer Hospital, and informed consent was waived because of the retrospective nature of this study.

### Transarterial Chemoembolization

Most of patients were treated with conventional TACE (cTACE) from 2000 to 2016, and some patients received drug-eluting bead (DEB) TACE from 2012 to 2016 (15). Patients underwent selective arteriography of the hepatic artery to define the locations of tumor. Once the tumor-nourishing artery was identified, the percutaneous femoral artery was punctured using the Seldinger technique. A 2.7F microcatheter was subsequently used for catheterization, and the chemotherapy drug solution (either pirarubicin 60–80 mg or cisplatin 80 mg/m^2^), normal lipiodol or ethiodized poppy seed oil (5–15 mL) as drug carriers, and blank CalliSpheres® microspheres (CSM) with diameters of 500 to 700, 300 to 500, or 100 to 300 μm as embolization agents were infused into the tumor supplying vessel. Finally, the angiography was checked for another time to ensure the normal lipiodol. For drug-loading process of DEB-TACE, the CSM with diameters of 100 to 300 μm was loaded with pirarubicin (60–80 mg) in the DEB-TACE procedures. In addition, for the massive HCCs, TACE was treated multiple times.

### Hypofractionated Conformal RT

Patients in the TACE + RT group underwent TACE followed by RT at intervals of 2 to 4 weeks. Patients with a hepatic arteriovenous fistula or other TACE contraindication received RT only. This technique has been described in a previously published article (16–18). Hypofractionated RT was delivered using a linear accelerator with three-dimensional conformal radiation therapy (3D-CRT) or intensity-modulated radiation therapy (IMRT). The gross tumor volume was defined by the hyperdense area of the intrahepatic primary tumor during the arterial phase and the hypodense filling defect area of the venous thrombus including PVTT, HVTT, and/or IVCTT during the venous phase. The clinical target volume (CTV) was determined with a 0.5-cm margin of the primary mass and 0.5-cm margin of the distal end of the venous thrombus. The planning target volume (PTV) was established by adding 1- to 1.5-cm margin to the CTV in the cranial–caudal axis and 0.5 cm in the anterior–posterior and lateral axes for uncertainties in treatment delivery.

Hypofractionation was applied every other day (three fractions a week). The fraction doses were decided on the basis of the following principle: 3–5 Gy per fraction for tumors larger than 5 cm in diameter and more than 5–6 Gy per fraction for tumors <5 cm in diameter. The median radiation dose finally delivered to the isocenter was 52 Gy (range, 36.5–64 Gy), with a median per dose of 4.5 Gy (range, 2–8 Gy), and fractions of 12 (range, 4–32 Gy) and in a median total irradiation time of 26 days (range, 8–57 days). The RT plan was evaluated by the dose volume histogram, and PTV was calculated to cover 95% of the isodose curve. In regard to the liver, the absolute normal liver volume (mL, total liver minus PTVs) was >700 mL. The mean dose to normal liver was <23 Gy, and/or the percentage normal liver volume receiving more than 20 Gy (V20) was <48.5%.

### Determination of Treatment Efficacy

Survival of the enrolled patients was reevaluated 1 month after treatment and subsequently every 3 or 6 months. Contrast-enhanced CT and/or MRI was compared at each follow-up visit by the treating radiation oncologist. Response Evaluation Criteria in Solid Tumors guideline was used to evaluate the changes of liver tumor. The presence of grade 3 or more severe early and late adverse events was investigated based on the Common Terminology Criteria for Adverse Events version 4.0.

### Propensity Score–Matching Analysis

Propensity score matching analysis was applied to adjust for potential treatment assignment imbalances. A 1:1 ratio matching between the RT and TACE + RT groups was performed to maximize the propensity score match with a caliper value of 0.3. Tumor size, type of MVI, ECOG, albumin–bilirubin (ALBI) score, age, gender, α-fetoprotein, and hepatitis B virus were selected on the basis of this propensity score.

### Statistical Analyses

Overall survival (OS) was defined as the time span between the date of treatment and the date of final follow-up or death. Progression-free survival (PFS) was evaluated from the date of treatment until the date of extrahepatic and/or intrahepatic disease progression or recurrence or death. Intrahepatic control (IC) was defined as no intrahepatic progression or recurrence of tumor tissue, including region outside of the radiation treatment field. Overall survival, PFS, and IC rate were calculated using the Kaplan–Meier method with the log-rank test. Student *t* and Mann-Whitney *U* tests were used to analyze continuous variables. For categorical variables, the χ^2^ test and Fisher exact test were performed. Univariate and multivariate analyses were performed using the Cox proportional hazards model. All statistical analyses were performed using R version 3.6.1 software (2019 Microsoft Corporation, USA). *P* < 0.05 was considered statistically significant.

## Results

### Baseline Characteristics of Patients

Patient characteristics are described in Table 1. One hundred forty-eight treatment-naive HCC patients with MVI were included in the present study. A total of 99 cases (63.6%) received RT only (RT group) and 49 cases (36.3%) were treated with TACE combined with RT (TACE + RT group). During follow-up, 126 patients (85.1%) died. The median follow-up time was 55.0 months in the RT group and 57.0 months in the TACE + RT group. Some variables differed between the groups, including ECOG, hepatitis B infection, ALBI score, and levels of albumin and alkaline phosphatase. After propensity score matching, 41 paired patients were selected from the RT and TACE + RT groups. Baseline characteristics were well-balanced between the two groups.

**Table 1:** Patient and treatment characteristics between the two groups.

		**Before propensity matching**			**After propensity matching**			
**Factor**	**Level**	**RT**	**TACE + RT**	***P***	**RT**	**TACE + RT**	***P***	**Absolute standardized difference**
*n*		99	49		41	41		
Gender	Male	88 (89%)	41 (84%)	0.37	37 (90%)	37 (90%)	1	0.63
	Female	11 (11%)	8 (16%)		4 (10%)	4 (10%)		
Age, median (IQR) (y)		47 (40, 55)	47 (41, 56)	0.61	44 (38, 50)	47 (41, 54)	0.42	−0.19
ECOG	0	8 (8%)	13 (27%)	0.002	6 (15%)	7 (17%)	0.76	0.758
	1	91 (92%)	36 (73%)		35 (85%)	34 (83%)		
Hepatitis B virus	Negative	21 (21%)	2 (4%)	0.007	3 (7%)	2 (5%)	0.64	0.633
	Positive	78 (79%)	47 (96%)		38 (93%)	39 (95%)		
KPS, median (IQR)		80 (80, 80)	80 (80, 90)	0.063	80 (80, 80)	80 (80, 80)	0.95	0.887
Tbil, median (IQR) umol/L		15.7 (12.3, 25.1)	15.2 (10.9, 19.2)	0.26	14 (10.8, 21.9)	15.7 (10.8, 19.2)	0.94	0.68
Albumin, median (IQR) g/L		37.8 (35, 40.3)	40 (38.8, 43)	<0.001	39.9 (37.3, 43.2)	39.6 (38.6, 42.8)	0.78	0.77
Urea, median (IQR) mmol/L		4.7 (3.9, 5.4)	5 (3.92, 6.5)	0.17	4.5 (3.6, 5.1)	4.78 (4, 6.71)	0.33	0.16
ALP, median (IQR) U/L		140 (108, 194)	116 (83, 172)	0.027	130 (96, 189)	132 (99, 173)	0.6	0.573
PT, median (IQR) sec		12.6 (12,14)	13 (12,14)	0.34	12.6 (12, 13.9)	12.8 (12,14)	0.51	0.17
ALBI score, median (IQR)		−2.47041 (−2.66009, −2.12306)	−2.6852 (−2.88263, −2.54889)	<0.001	−2.5828 (−2.85849, −2.34022)	−2.6714 (−2.80294, −2.485)	0.69	0.68
ALBI grade, median (IQR)		2 (1,2)	1 (1,2)	<0.001	2 (1,2)	1 (1,2)	0.27	0.26
AFP, median (IQR) ng/ml		450 (21.74, 1480)	259.1 (30, 600)	0.23	450 (18, 1400)	290 (10, 600)	0.24	0.01
Tumor size, median (IQR), cm		9 (7, 11.2)	7.8 (5.5, 11.2)	0.2	9 (7, 10.5)	8 (6, 11.5)	0.63	0.43
Tumor number	1	81 (82%)	42 (86%)	0.82	33 (80%)	35 (85%)	0.8	−0.02
	2	6 (6%)	2 (4%)		2 (5%)	2 (5%)		
	≥3	12 (12%)	5 (10%)		6 (15%)	4 (10%)		
Cheng's type of PVTT	I	3 (3%)	3 (6%)	0.35	2 (5%)	2 (5%)	0.23	−0.12
	IIa	56 (57%)	28 (57%)		23 (56%)	25 (61%)		
	IIb	5 (5%)	1 (2%)		2 (5%)	1 (2%)		
	III	27 (27%)	11 (22%)		12 (29%)	9 (22%)		
	IV	3 (3%)	0 (0%)		2 (5%)	0 (0%)		
	IVCTT	5 (5%)	6 (12%)		0 (0%)	4 (10%)		
RT dose, median (IQR), Gy		52 (48, 55.2)	49 (45, 54)	0.38	52 (45, 56)	49.5 (46.4, 54)	0.62	0.24
RT fractions median (IQR)		12 (10,14)	12 (10,15)	0.88	12 (10,15)	11 (10,15)	0.58	−0.3

*AFP, α-fetoprotein; ALBI, albumin–bilirubin; ALP, alkaline phosphatase; IQR, interquartile range; PT, prothrombin time; RT, radiation therapy; TACE, transarterial embolization*.

### TACE + RT vs. RT

**Figure 1:**
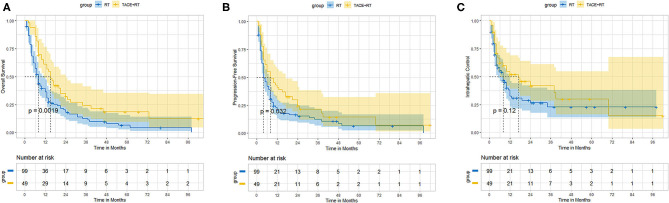
Before propensity matching, the TACE + RT group vs. the RT group. **(A)** Overall survival, **(B)** progression-free survival, **(C)** intrahepatic control.

Before propensity score matching, the TACE + RT group exhibited better OS and PFS than did the RT group. The median OS was better in the TACE + RT group than RT group (15.0 vs. 8.0 months, *P* < 0.001). The 1-, 2-, 3-, and 5-years OS rates in the TACE + RT group were 61.0%%, 32.2%, 26.8%, and 18.4% vs. 36.2%, 17.9%, 12.4%, and 4.3% in the RT group, respectively (*P* = 0.0019; Figure 1A). The median PFS was better in the TACE + RT group than the RT group (8.0 vs. 4.0 months, *P* = 0.01). The 6-, 12-, and 24-months rates in the TACE + RT group were 55.2%%, 40.8%, and 23.0% vs. 41.9%, 19.9%, and 15.2% in the RT group, respectively (*P* = 0.032; Figure 1B). Nevertheless, the two groups were comparable in IC rate (*P* = 0.12; Figure 1C).

**Figure 2:**
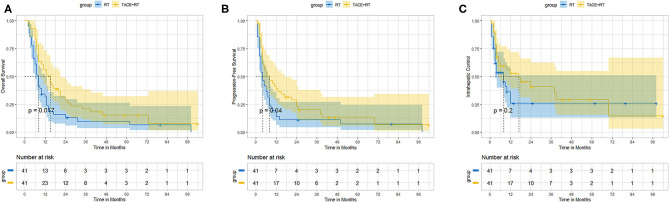
After propensity matching, the TACE + RT group vs. the RT group. **(A)** Overall survival, **(B)** progression-free survival, **(C)** intrahepatic control.

Of 41 cases well-pairs after propensity score matching, TACE + RT also had better OS and PFS than the RT group. The median OS was also better in the TACE + RT group than the RT group (15.0 vs. 8.0 months, *P* = 0.017). The 1-, 2-, 3-, and 5-years OS rates in the TACE + RT group were 56.1%%, 28.6%, 20.8%, and 15.7% vs. 31.5%, 13.1%, 9.8%, and 6.7% in the RT group, respectively (*P* = 0.017; Figure 2A). The median PFS was better in the TACE + RT group than in the RT group (8.0 vs. 4.0 months, *P* = 0.01). The 6-, 12-, and 24-months rates in the TACE + RT group were 51.2%, 39.0%, and 23.1% vs. 36.6%, 13.9%, and 11.1% in the RT group, respectively (*P* = 0.04; Figure 2B). Nevertheless, the two groups were comparable in IC rate (*P* = 0.20; Figure 2C).

Univariate and multivariate analyses were performed by using the Cox proportional hazards model (Table 2); TACE (no/yes) [*P* = 0.037; hazard ratio (HR) = 0.648; 95% confidence interval (CI) = 0.43–0.975], KPS score (*P* = 0.019; HR = 0.965; 95% CI = 0.937–0.994), and gender (male/female) (*P* = 0.005; HR = 0.381; 95% CI = 0.193–0.75) were three independent predictors of OS. The included influencing factors were not prognostic factors for PFS. Gender (male/female) (*P* = 0.04; HR = 0.443; 95% CI = 0.204–0.962) was an independent predictor of IC.

**Table 2:** Prognostic factors for IC, PFS, and OS based on univariate and multivariate analyses.

		**IC**						**PFS**						**OS**					
		**Univariate**			**Multivariate**			**Univariate**			**Multivariate**			**Univariate**			**Multivariate**		
**Factors**	**Level**	**P**	**HR**	**95%CI**	**P**	**HR**	**95%CI**	**P**	**HR**	**95%CI**	**P**	**HR**	**95%CI**	**P**	**HR**	**95%CI**	**P**	**HR**	**95%CI**
Gender	Male																		
	Female	0.035	0.435	0.2–0.944	0.04	0.443	0.204–0.962	0.084	0.612	0.35–1.068				0.005	0.421	0.232–0.767	0.005	0.381	0.193–0.75
Age, median (IQR)		0.192	0.987	0.969–1.006				0.297	0.992	0.976–1.007				0.579	0.995	0.98–1.012			
ECOG	0																		
	1	0.463	1.257	0.682–2.316				0.381	1.25	0.758–2.062				0.475	1.206	0.722–2.015			
Hepatitis B virus	Negative																		
	Positive	0.503	1.224	0.678–2.21				0.357	1.253	0.775–2.025				0.29	1.303	0.798–2.128			
KPS, median (IQR)		0.222	0.98	0.948–1.013				0.058	0.973	0.946–1.001				0.028	0.968	0.941–0.996	0.019	0.965	0.937–0.994
Tbil, median (IQR)		0.669	1.001	0.995–1.007				0.53	1.002	0.997–1.007				0.014	1.006	1.001–1.01	0.418	1.004	0.995–1.013
Albumin, median (IQR)		0.054	0.949	0.899–1.001				0.037	0.954	0.913–0.997	0.464	0.966	0.882–1.059	0.006	0.939	0.898–0.982	0.501	0.948	0.811–1.108
Tumor size, median (IQR)		0.705	1.013	0.948–1.082				0.886	1.004	0.95–1.061				0.516	1.018	0.964–1.076			
Urea, median (IQR)		0.913	0.992	0.862–1.142				0.729	0.979	0.87–1.102				0.708	0.978	0.868–1.101			
ALP median (IQR)		0.878	1	0.998–1.002				0.191	1.001	1–1.002				0.034	1.001	1–1.002	0.218	1.001	0.999–1.002
PT, median (IQR)		0.651	1.03	0.907–1.169				0.969	1.002	0.899–1.117				0.63	1.028	0.919–1.149			
ALBI score, median (IQR)		0.068	1.641	0.964–2.795				0.045	1.574	1.011–2.452	0.931	1.043	0.402–2.705	0.001	2.087	1.347–3.236	0.731	0.735	0.128–4.235
AFP, median (IQR)		0.16	1	43831				0.099	1	43831				0.015	1	1.0–1.0	0.087	1	1.0–1.0
RT dose, median (IQR)		0.094	0.975	0.947–1.004				0.133	0.981	0.957–1.006				0.09	0.978	0.953–1.003	0.073	0.977	0.953–1.002
Tumor number	1	0.93						0.931						0.865					
	2	0.749	0.862	0.348–2.136				0.747	0.881	0.409–1.898				0.653	0.828	0.363–1.887			
	≥3	0.815	0.92	0.459–1.844				0.823	0.936	0.525–1.67				0.794	1.081	0.604–1.934		
Cheng's type of PVTT	I	0.749						0.595						0.308					
	IIa	0.921	1.061	0.329–3.419				0.821	0.9	0.362–2.24				0.653	0.811	0.325–2.023			
	IIb	0.915	0.906	0.151–5.448				0.373	1.719	0.522–5.665				0.382	1.699	0.517–5.58			
	III	0.557	1.431	0.433–4.731				0.938	1.038	0.405–2.662				0.717	0.839	0.325–2.168			
	IV	0.472	1.932	0.322–11.609				0.428	1.786	0.425–7.504				0.305	2.125	0.503–8.971			
	IVCTT	0.846	0.872	0.218–3.492				0.744	0.836	0.285–2.452				0.452	0.656	0.218–1.97			
Child–Pugh	A																		
	B	0.882	1.061	0.489–2.302				0.472	1.244	0.686–2.258				0.005	2.23	1.269–3.921	0.594	1.259	0.541–2.928
TACE	No																		
	Yes	0.137	0.709	0.451–1.115	0.164	0.725	0.46–1.14	0.044	0.679	0.466–0.989	0.123	0.735	0.497–1.087	0.003	0.56	0.382–0.821	0.037	0.648	0.43–0.975

### Recurrence

During the follow-up period, intrahepatic recurrence was observed in 15 cases (30.6%) in the TACE + RT group and 21 cases (21.2%) in the RT group. The extrahepatic metastasis was observed in seven cases (14.3%), including three lung metastases, three abdominal aortic lymph node metastases, and one bone metastases in the TACE + RT group, and 13 (13.1%) cases including 10 lung metastases, three abdominal aortic lymph node metastases, and one adrenal metastasis in the RT group.

### Complications

Eleven patients (7.4%) experienced radiation-induced liver disease (RILD) among all patients, with five (35.7%) of Child–Pugh B and six (4.5%) of A class, including two (4.1%) in the TACE + RT group and nine (9.1%) patients in the RT group, respectively. Five patients with RILD died within 1–2 months, and two died within 5–6 months after RT treatment.

One (2.0%) experienced RT-related gastrointestinal (GI) bleed in the TACE + RT groups and two (2.0%) in the RT groups. Three cases (3.0%) experienced hepatic encephalopathy in the RT group.

The most common acute toxicities (1–2 grade) including fatigue, anorexia, nausea, and/or radiation dermatitis were observed in 60 patients (60.0%) of the RT group and in 31 patients (63.2%) of the RT + TACE group, respectively. These complications were successfully managed by conservative treatments.

## Discussion

Major vessel invasion is a terminal manifestation of HCC, carries an extremely poor prognosis with a median survival of only 4.0–5.2 months in symptomatic supportive treatment (19, 20). In the current study of hepatitis B virus–related HCC, the TACE + RT group exhibited better OS (15.0 vs. 8.0 months) and PFS (8.0 vs. 4.0 months) than did the RT group. The responders of primary tumor and/or thrombosis could have significantly better survival than non-responders in the published literature (21–23). In current study, we were unable to fully assess the primary tumor or thrombosis response during long-term follow-up. Although IC including in- and out-field-treated (PTV) lesions in the whole liver did not achieve a significant statistical difference (median IC = 17.0 vs. 8.0 months), the two curves tended to separate. On multivariate analysis, treatment option (TACE + RT vs. RT) was a significant covariate associated with OS (*P* = 0.037; HR = 0.648; 95% CI = 0.43–0.975).

Over the past decade, sorafenib is recommended as a first-line treatment for patients with advanced liver cancer in BCLC-C stage (6, 24, 25). But sorafenib therapy for HCC with MVI extended survival somewhat disappointingly by 4.0 months (8.9 vs. 4.9 months) in subanalyses of a phase III SHARP trial in a western country (26), whereas it was only 1.5 months in HCC in an Asia-Pacific trial (5.6 vs. 4.1 months) (27). The objective response rate of 2.0 to 3.3% in patients treated with sorafenib warrants a better treatment modality (24, 25). Radiotherapy plays an increasingly important role in the treatment of MVI (10, 13, 28, 29). Several retrospective analyses showed that RT was preferable to surgery in patients with [Cheng's classification (30)] type III PVTT, and similar outcome in the type II PVTT group, but lower OS in the type I PVTT group (31, 32). A randomized clinical trial in South Korea first suggested that TACE + RT provided an improved PFS, objective response, and OS compared with sorafenib treatment for patients with locally advanced HCC with MVI (10). In this retrospective study, our result demonstrated that the combined treatment of TACE followed by RT provided a better PFS and OS compared with RT alone.

The results following RT alone or TACE and RT were better compared to untreated controls or those treated with TACE alone (11–14). Radiotherapy may prolong survival in patients with PVTT by achieving of 61.5% objective response rate, and 11% of patients became resectable after RT with a median survival of 30 months and a 2-years OS rate of 67% (33). Transarterial chemoembolization + RT also provided a chance of downstaging for curative resection in advanced patients with MVI and had better long-term survival (10). Chong et al. (34) reported that in the concurrent chemoradiotherapy (CCRT) followed by hepatic arterial infusion chemotherapy (HAIC) group, 26 (26.5%) of 98 patients downstaged and underwent subsequent curative resection. Disease-specific survival improved significantly in the resection after localized downstaging group than the resection-first group (median, 62 vs. 15 months, respectively; *P* = 0.006) (34). Lee et al. (35) also reported that 41 (16.9%) underwent curative resection after CCRT followed by HAIC and tumor downstaging of 32 (78%) of the resected patients. The 5-years survival of the curative resection group after CCRT was significantly higher than that of the CCRT-alone group (49.6% vs. 9.8%; *P* < 0.001) (35). Wei et al. (29) reported that neoadjuvant RT (3 Gy × 6 fractions = 18 Gy) provided significantly better postoperative survival outcomes in type II/III PVTT than surgery alone in a randomized, multicenter controlled trial, in which 20.7% (17 of 82) of patients had partial response in the neoadjuvant RT group, and the OS rates for the neoadjuvant RT group at 12 and 24 months were 75.2 and 27.4% compared with 43.1 and 9.4% (*P* < 0.001) in the surgery-alone group, respectively (29).

Historically, 2D-CRT has made limited contributions to the treatment of HCC because of the high incidence of RILD (36). Despite advances in RT delivery including 3D-CRT, IMRT, volumetric modulated arc therapy, and stereotactic RT (SBRT), hepatic toxicity following RT remains a dose-limiting complication (37). In current study, 7.4% (11 of 148) of patients experienced RILD with 35.7% of Child–Pugh B and 4.5% of A class, indicating that patients with Child–Pugh A have better tolerance than B class, and TACE + RT had well-complementarity with no more complications than RT alone. Xu et al. reported that the hepatic tolerable doses (TD5) of mean dose to normal liver were 21 Gy and 6 Gy for Child–Pugh A and B patients, respectively (38). Liang et al. (16, 17) also reported the mean dose to normal liver at <23 Gy and/or V20 <48.5% could improve the safety of hypofractionated RT for primary liver carcinoma. In addition, a growing research has confirmed that SBRT was less likely to cause RILD (39).

The sequential order of the two schemes may affect the outcome and merits further study. Li et al. (40) found that IMRT + TACE had better survival outcomes and liver function when compared to TACE+IMRT for HCC with main trunk PVTT, but not HCC with only portal branch tumor thrombosis. We used to implement the protocol of TACE followed by RT instead of RT + TACE based on two aspects: (a) the short treatment time of TACE (1 day) and longer treatment time of RT (>20 days) make it convenient for combined application; (b) the timing of TACE treatment was uncertain after RT. It was feared that TACE may increase RILD because the adverse effects of RT were not relatively well-understood and controlled. In current study, we found that TACE + RT had well-complementarity with no more complications than RT alone.

There are some limitations to the present study. First, this study adopted a single-center retrospective design. Second, the long-term survival outcomes (5-years OS: 18.4 vs.4.3%) were obtained based on 85% of deaths of enrolled patients, but selection bias may have increased owing to the time span from 2000 to 2016. The protocols of TACE were performed with cTACE or DEB-TACE in recent years, and OS and PFS in DEB-TACE treatment were equivalent compared to cTACE (15). Meanwhile, the protocols of hypofractionated conformal RT have not changed much by using 3D-CRT or IMRT technology. We applied propensity score matching as an additional means to reduce selection bias, and the associations between TACE + RT and better OS and PFS remained after matching. Third, this study was performed in southern China where hepatitis B virus–related HCC is endemic; it is unclear whether the dosimetric findings are applicable to cases of HCC associated with other risk factors.

In conclusion, combined TACE and RT had well-complementarity with no more complications, providing a better PFS and OS compared with RT-alone treatment. A randomized prospective study is still needed to investigate the true effect.

## Data Availability Statement

The raw data supporting the conclusions of this article will be made available by Ting-Shi Su (sutingshi@163.com), without undue reservation.

## Ethics Statement

The studies involving human participants were reviewed and approved by the ethics review board of Guangxi Medical University Cancer Hospital (LW2020044). The ethics committee waived the requirement of written informed consent for participation.

## Author Contributions

T-SS and Li-QL made contributions to the study, data analysis, and interpretation and participated in the drafting of the article. T-SS, S-XL, and Le-QL made substantial contributions to the study's conception and design. All authors made substantial contributions to the acquisition of data and provided final approval of the version to be published.

## Conflict of Interest

The authors declare that the research was conducted in the absence of any commercial or financial relationships that could be construed as a potential conflict of interest.
